# O065: Using adenosine tri-phosphate (ATP)-bioluminescence assay to compare outcomes of two strategies to perform environmental cleaning in a hospital setting

**DOI:** 10.1186/2047-2994-2-S1-O65

**Published:** 2013-06-20

**Authors:** R Nativ, I Livshiz Riven, A Borer

**Affiliations:** 1 Infection Control Unit, Sorok University Medical Center, Israel; 2 Nursing, Ben Gurion of the Negev, Israel; 3 Infection Control Unit, Soroka University Medical Center, Beer-Sheva, Israel

## Introduction

Hospital environment poses a substantial risk for transmission of pathogens. However, assessing cleaning efficiency is difficult and often is carried out by a subjective visual check only. The ATP-bioluminescence assay technology was developed to assist in evaluation of the environmental cleaning process by counting living and non-living organic matter in Relative Light Units (RLUs). In a 1000-bed university-affiliated hospital the standard practice following patient discharge includes terminal cleaning of the patient unit performed by nursing aides associated with the ward.

## Objectives

The purpose of the study was to compare, in RLUs, the outcome of environmental and terminal cleaning in hospital wards using dedicated trained maintenance personnel (strategy 1) versus routine departmental cleaning (strategy 2). An additional purpose was to evaluate the applicability of the ATP-bioluminescence assay technology.

## Methods

An experimental design with an intervention (strategy 1) and control (strategy 2) was conducted on four comparable medical wards. Two wards used cleaning strategy 1 for terminal cleaning and the other two used cleaning strategy 2 as controls. Ten sites were examined in each ward with ATP-bioluminescence assay: 6 sites of high risk patient contact areas 30 minutes following terminal cleaning, and 4 sites for general environmental surroundings.

## Results

770 samples were collected; 373 from the intervention group wards and 397 from the control group wards. A statistically significant difference was found in 2 out of 6 high risk patient contact sites in the patient units: 1) the bed rails, with a median of 397 RLUs in the intervention group vs. 752 RLUs in the control group (*p*<0.001); and 2) the call button, with medians of 338 and 535 RLUs in the intervention and control group, respectively (*p*<0.002).

## Conclusion

Assigning a dedicated and trained team for terminal cleaning as a cleaning strategy demonstrated superiority at least in 2/6 high touch surface areas. The ATP-bioluminescence assay technology is a convenient way to check general cleanliness.

## Disclosure of interest

R. Nativ Grant/Research support from M3 Israel and CHEMITEC, I. Livshiz Riven: None declared, A. Borer: None declared.

